# An Address on the Orthopedic Outlook in Military Surgery

**Published:** 1918

**Authors:** 


### ABSTRACTS

### ORTHOPEDICS

***An Address on the Orthopedic Outlook in Military Surgery.*** By Colonel Sir ROBERT JONES, C. B. Ch. M., Inspector of Military Orthopedics, Army Medical Service. Abs. from *Brit. Med. Jour.*, Jan. 12, 1918.

The conditions which, taken together, create an orthopedic case may be classed roughly under the following heads :

1. The mechanical injury to bone, joint, muscle or nerve.
2. The atrophy and disease of these structures primarily due to the injury.
3. Incoordination of movement due to disease of the brain — a result of atrophy and disease of peripheral structures.
4. Psychological conditions which can be overcome by re-educational processes.

There are now sixteen centers in the British Isles containing close upon 15,000 wounded. Of the men treated in them 75 o/o have been returned to the army. An orthopedic center consists of :

1. A staff of surgeons who have had previous experience of the detail of orthopedic work, both operative, manipulative, and educational. They plan the complete course of treatment.
2. Men with experience in operative surgery who, though not specializing in the work, are interested in it, and only need experience to fit them to take charge of wards in new centers as they are formed.
3. Still younger men who will ultimately go abroad, to whom the educational advantages offered by the centers is of the greatest benefit.
4. A series of auxiliary departments, each under a medical man who has experience of the particular methods of the treatment he directs. These departments are the electric, the massage, the hydrological, the gymnastic and the curative workshops.

The successful working of an orthopedic center depends upon the coherent association of all departments in carrying out the plan of campaign mapped out by the surgeon when he sees the case.

Workshops have proved of considerable value, particularly from a disciplinary point of view. The work done in them — splint making, boot repairing, electrical fitting adjustment, clerical work, etc. — is productive and useful, must be performed by someone, and the wounded man feels that if it is worth doing at all it is worth doing well. In prescribing the work, however, care must be taken not to fatigue the disabled limb; indirect use gives better results, and the member should be exercised daily in one of the special departments.

The orthopedic problem can be divided into two distinct parts — preventive orthopedics and corrective orthopedics. The question of fractures of the femur is essentially one of *preventive orthopedics.* All fractured femurs should be concentrated in special fracture hospitals at the base. Each hospital should be staffed by well-trained surgeons with mechanical aptitude, desirous of devoting themselves to this special work for the term of their service. They should have selected nurses and orderlies to help them in team work, and they should be protected from the danger of frequent changes. Two fundamental principles must be sacredly adhered to:

1. Efficient fixation in correct alignment at the earliest possible moment.

2. Continuity of treatment.

For the purpose of rapid, simple and efficient fixation there is no splint to compare with a Thomas, which can be applied over the trousers and extension made by a pull on the boot. Cases should be sent down from the casualty clearing stations at the earliest possible moment after recovery from shock, before sepsis has had time to spread and before the danger of secondary hemorrhage has set in; they do best at the base hospitals when they are received there during the first three days. They should then go on to orthopedic hospitals, where it must be remembered that gunshot fractures of bones take a considerably longer time to harden than those encountered in civilian practice.

Both at home and abroad the defects of treatment run on similar lines — namely, inefficient reduction, fixation, and extension. What has been said about fracture of the femur is true of fractures of other bones. In the lower limb, fractures are too often associated with a backward sagging at the site of fracture, due to inefficient support; or they present a valgus deformity which renders the leg both weak and ugly. In fractures of the humerus, nonunion, or delayed union, is far too common, particularly when the fracture is through the middle third; this is mainly due to two factors — over-extension and inefficient fixation. The prevailing deformity in fractures of the lower end of the humerus is a backward thrust of the elbow. This is best governed by flexing the elbow sufficiently. Fractures through the elbow-joint, in view of ankylosis, should be treated at right angles. In this form of fracture, care should be taken to keep the forearm about three-fourths supinated, for a pronated hand with ankylosed elbow is tragic. To prevent vicious union between the two bones, and sagging or convexity to the ulnar side, the ulna must be placed in line before supination is secured. It is a sound rule in all fractures of the upper limb to see that *the palm is towards the face when the elbow is flexed.*

The functional results of primary excision of joints, as they are now filtering into the orthopedic centres, are very bad. On the other hand the results of immediate excision of the *wound* (not the joint) in cases of gunshot injuries to articulations, are often surprisingly good. A few suggestions may be offered from the orthopedic standpoint as to the after-treatment of these excisions :

If the shoulder is excised, the arm should be placed in an abducted position at an angle of about 50 degrees. The elbow should be slightly in front of the coronal plane of the body, so that when it is at right angles and the forearm supinated, the palm of the hand is towards the face. The bones should be approximated by posture at an early date. If ankylosis occurs in this position, the arm can be lifted to a considerable height by scapular action. The arm should not be left hanging, as there is on one side danger of a useless flail shoulder, while on the other, should it become ankylosed, the movement will be very limited. If the excision should result in a flail shoulder, it should be ankylosed in the position just described.

The same principles govern post-operative treatment of the elbow. After being extended and supinated during the early stages of drainage, it should be gradually flexed to a right angle, even if sinuses discharge; as, if it is kept straight while a suppurating wound heals, there is a danger of ankylosis in extension. If there is no suppuration it can be flexed from the first.

When ankylosis is feared, a joint should be placed in the position most useful for the patient in after-life : the shoulder abducted as described, the elbow at an angle of about 70° from full extension, the forearm nearly two-thirds supinated, the wrist in dorsiflexion, the hip in slight abduction and external rotation with full extension, the knee in full extension, the ankle at right angles and very slightly varoid.

The importance of position becomes evident again in injuries of muscles and nerves. Voluntary action of the muscle — not necessarily movement of the limb — must be encouraged at the earliest stage. The surgeon must, however, watch that contracture or over-stretching does not take place. In recovering nerve injuries, the muscles supplied by the nerve must be kept continuously relaxed by splinting the limb. All deformities which would impede the action of the muscles should be corrected as a preliminary to nerve suture unless this subsequent correction can be easily secured without straining the fresh union in the nerve.

In corrective orthopedic surgery, the treatment of stiff joints presents a whole series of problems. A surgeon without considerable experience of manipulative methods, when tempted to move a stiff joint after gunshot injuries, had better pause, reflect, and refrain. This should be emphasized because in so many cases joints have been forcibly moved with disastrous results. If a stiff joint has to be moved under an anesthetic the bones above and below the joint must be protected, especially where a fracture has existed.

The orthopedic mind strives for function rather than for form, but is more content if it can secure excellence in both; it is conservative and constructive, but it desires to take the most direct road to function — be it by knife, by hand, or by suasion.

